# DNA Self-assembly Catalyzed by Artificial Agents

**DOI:** 10.1038/s41598-017-07210-y

**Published:** 2017-07-28

**Authors:** Chao Shi, Yifan Wang, Menghua Zhang, Cuiping Ma

**Affiliations:** 1 College of Life Sciences, Qingdao University, Qingdao, 266071 P.R. China; 2 Key Laboratory of Sensor Analysis of Tumor Marker, Ministry of Education, College of Marine Science and Biological Engineering, Qingdao University of Science and Technology, Qingdao, 266042 P.R. China

## Abstract

Nucleic acids have been shown to be versatile molecules and engineered to produce various nanostructures. However, the poor rate of these uncatalyzed nucleic acid reactions has restricted the development and applications. Herein, we reported a novel finding that DNA self-assembly could be nonenzymatically catalyzed by artificial agents with an increasing dissociation rate constant K2. The catalytic role of several artificial agents in DNA self-assembly was verified by real-time fluorescent detection or agarose gel electrophoresis. We found that 20% PEG 200 could significantly catalyze DNA self-assembly and increase the reaction efficiency, such as linear hybridization chain reaction (HCR) and exponential hairpin assembly (EHA). Therefore, we foresee that a fast and efficient DNA self-assembly in structural DNA nanotechnology will be desirable.

## Introduction

Nucleic acids have indeed been employed as an intelligent material for engineering molecular devices^1, 2^, walkers^3, 4^, DNA circuits^5, 6^, DNA origami^7, 8^ and amplifiers^9, 10^, which have therefore attracted more and more interest among researchers. A key feature of the nanostructures is that they do not require protein enzymes, and only require base-pairing between nucleic acid strands^11^. However, the rate of these uncatalyzed reactions is relatively poor. For example, the hybridization chain reaction (HCR) based on two stable species of DNA hairpins produces a linear growth polymerization chain reaction, which even was incubated for 24 h at room temperature^12^. How to increase the rate and yield of these nanostructured assembly will be very crucial for future developments within this field.

According to the results of exploring isothermal nucleic acid amplification methods, we found that betaine was able to inhibit the reaction efficiency of isothermal nucleic acid amplification^13^. The further researches showed that although betaine can lower the melting template (Tm) of DNA, betaine can decrease association rate constant K1. Betaine acted as a molecular barrier to intermolecular hybridization for isothermal nucleic acid amplification, resulting in lowering the reaction efficiency^13^. The finding has inspired our interest in the subject. Polyethylene glycol (PEG) and Cetyltrimethylammonium Bromide (CTAB) have been reported to improve the rate of DNA hybridization, so we guessed they might promote DNA self-assembly reaction^14, 15^. We here mainly studied the effect of PEG on DNA self-assembly reaction. According to our finding, we inferred that those substances lowering the Tm of DNA and increasing dissociation rate constant K2 should be more beneficial to increase the dissociation degree of DNA and stimulate DNA hybridization reaction.

## Materials and Methods

### Materials

All nucleic acids used in this work (Supplementary Table S1) were designed by using NUPACK software (http://www.nupack.org/) and produced by Shanghai Sangon Bio-Engineering Company (Shanghai, China). Betaine was purchased from Ourchem (Shanghai, China). CTAB was purchased from Solarbio (Beijing, China). Pullulan, Dithiothreitol (DTT), Dimethyl sulfoxide (DMSO), PEG 200, PEG 600, PEG 2000 and PEG 6000 were purchased from Aladdin Industrial Corporation (Shanghai, China). 20 × Eva Green was purchased from Bridgen (Beijing, China). 2000 bp DNA Marker and the chemicals used to prepare electrophoresis were purchased from Dalian Takara Company (China).

## Methods

### DNA strand exchange reaction

The A^F^B^D^ double-stranded DNA (dsDNA) was obtained by mixing individual strand in 95 °C for 5 min, then cooling slowly from 95 °C to room temperature. The A^F^B^D^ dsDNA (4.0 × 10^−7^ M) was incubated at 37 °C with ssDNA B (2.0 × 10^−6^ M) in 1× Thermopol Buffer (20 mM Tris-HCl, 10 mM KCl, 10 mM (NH_4_)_2_SO_4_, 2 mM MgSO_4_ and 0.1% Triton X-100, pH 8.8) in the absence or presence of different artificial agents.

### EHA reaction

The EHA reaction system contained four hairpins H1, H2, H3, and H4^16, 17^. Here, the final concentrations of H1, H2, H3 and H4 were all 5.0 × 10^−7^ M (10 mM PBS, 5 mM MgCl_2_, pH 7.4). Different concentrations of PEG 200 were added to a final volume 100 μL, followed by the real-time fluorescence detection performed using a Hitachi F-4500 spectrophotometer (Tokyo, Japan) equipped with a xenon lamp at 1 min intervals. The reaction system was incubated at room temperature for 10 min, and directly subjected to 2% agarose gel electrophoresis, which was carried out in tris-acetate-EDTA (TAE) buffer at 110 V constant voltage for 15 minutes.

## Results and Discussion

### The effect of some substances on strand exchange reaction

**Figure 1:**
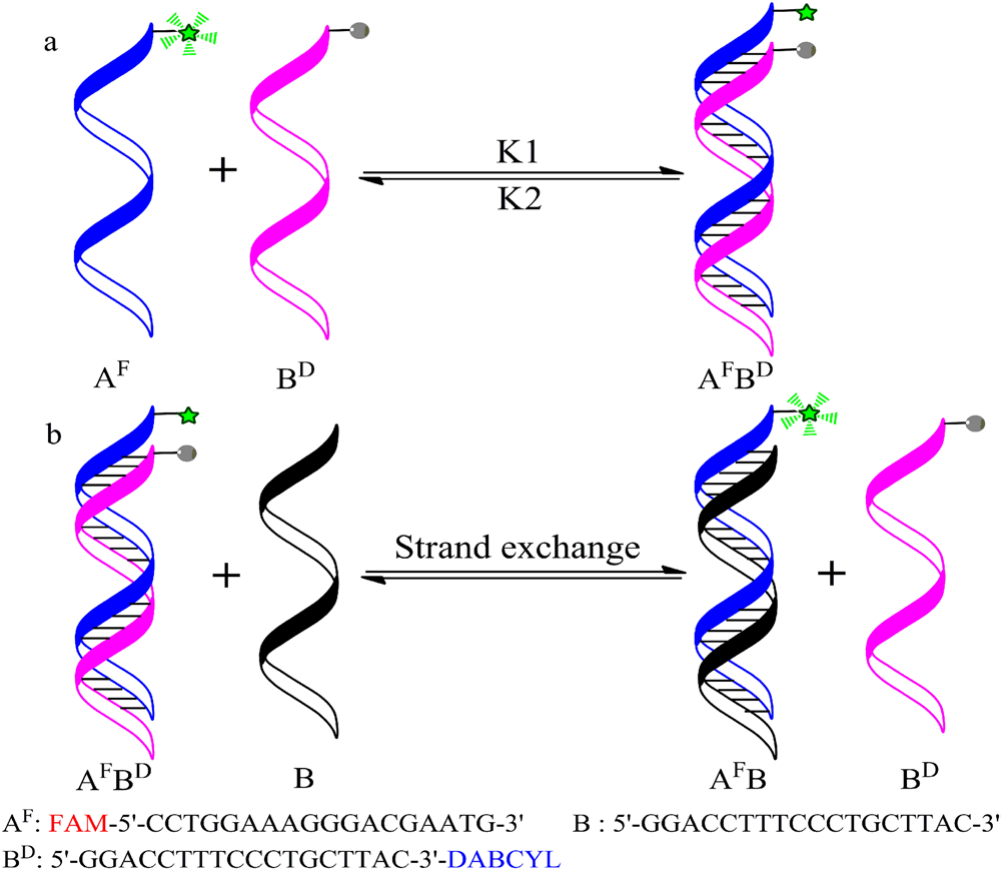
(**a**) Experimental schematic of DNA hybridization. (**b**) The principle of DNA strand exchange detection by using fluorescence resonance energy transfer (FRET) and the oligonucleotide sequences used. Two DNA strands A^F^ and B^D^ were respectively labeled with fluorescein at the 5′-end and DABCYL at the 3′-end. A^F^ was complementary to B or B^D^. The fluorescence emission increased when B^D^ strand was exchanged by B, due to the dissociation of FAM-DABCYL FRET pair.

**Figure 2:**
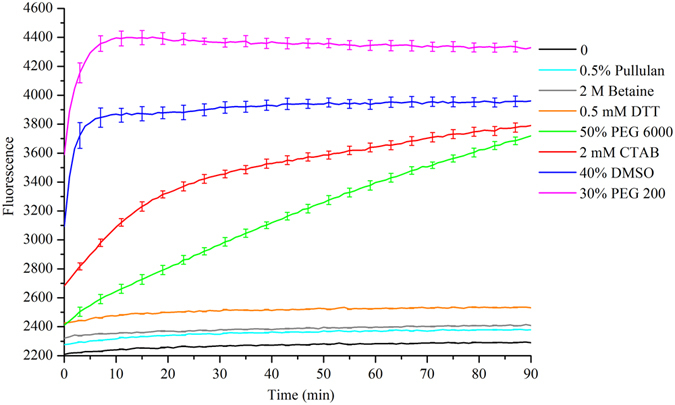
The comparison of the effects of several substances on DNA strand exchange by real-time fluorescence.

When dissociation rate constant K2 increased, unwinding of A^F^B^D^ was prone to happen (Fig. 1a and Supplementary Figure S1). B^D^ strand was exchanged by a single-stranded DNA (ssDNA) B resulting in fluorescence emission increased (Fig. 1b). At the same time, the fluorescence signal of reaction system with 30% PEG 200 rapidly increased compared with reaction system without PEG 200. This implied that PEG 200 indeed could unwind double-stranded DNA, and increased dissociation rate constant K2 (Supplementary Figure S1). So, we conducted a systematic review of the available literature and found some substances that may accelerate DNA strand exchange and increase dissociation rate constant K2^18–24^. The effects of different concentrations of these substances on DNA strand exchange were investigated and the results were shown in Supplementary Figures S2–10. Their optimal concentrations for DNA strand exchange were further compared with each other (Fig. 2). Only a slight increase in FAM fluorescence intensity was observed compared with the additive-free system, when 0.5% pullulan, 2 M betaine, and 0.5 mM DTT were respectively employed as an additive to DNA strand exchange. Moreover, there was no increase of fluorescence intensity in a time dependent manner. These results confirmed 0.5% pullulan, 2 M betaine, and 0.5 mM DTT had a little impact on DNA strand exchange. But a big jump in fluorescence intensity was observed compared with the additive-free system, when 30% PEG 200, 40% DMSO, 2 mM CTAB and 50% PEG 6000 were respectively added to reaction system. Notably, the fluorescence intensity was rapidly increased within 10 min, when 30% PEG 200 and 40% DMSO were respectively added, and an accelerated effect of 30% PEG 200 was greater than that of 40% DMSO. We inferred that the artificial agents increasing dissociation rate constant K2, such as PEG 200, could promote DNA strand exchange reaction by a concentration dependent manner. The reason that PEG200 could accelerate DNA strand exchange reaction may be molecular crowding^25, 26^ and hydrophobic interactions^20^.

The rate constant of DNA strand exchange reaction (SER) was determined by fitting the experimental data to a first-order reaction equation: (A_t_ − A_0_) = (A_∞_ − A_0_) (1 − exp (−kt)) where t is time, and A_t_, A_0_ and A_∞_ are the fluorescence intensity at time t, zero time and infinite time, respectively^27^. And the rate constant of SER added 30% PEG 200 was about 1000 folds compared with that of PEG 200-free SER. Thereby, PEG 200 was chose for further research.

### HCR catalyzed by PEG 200

**Figure 3:**
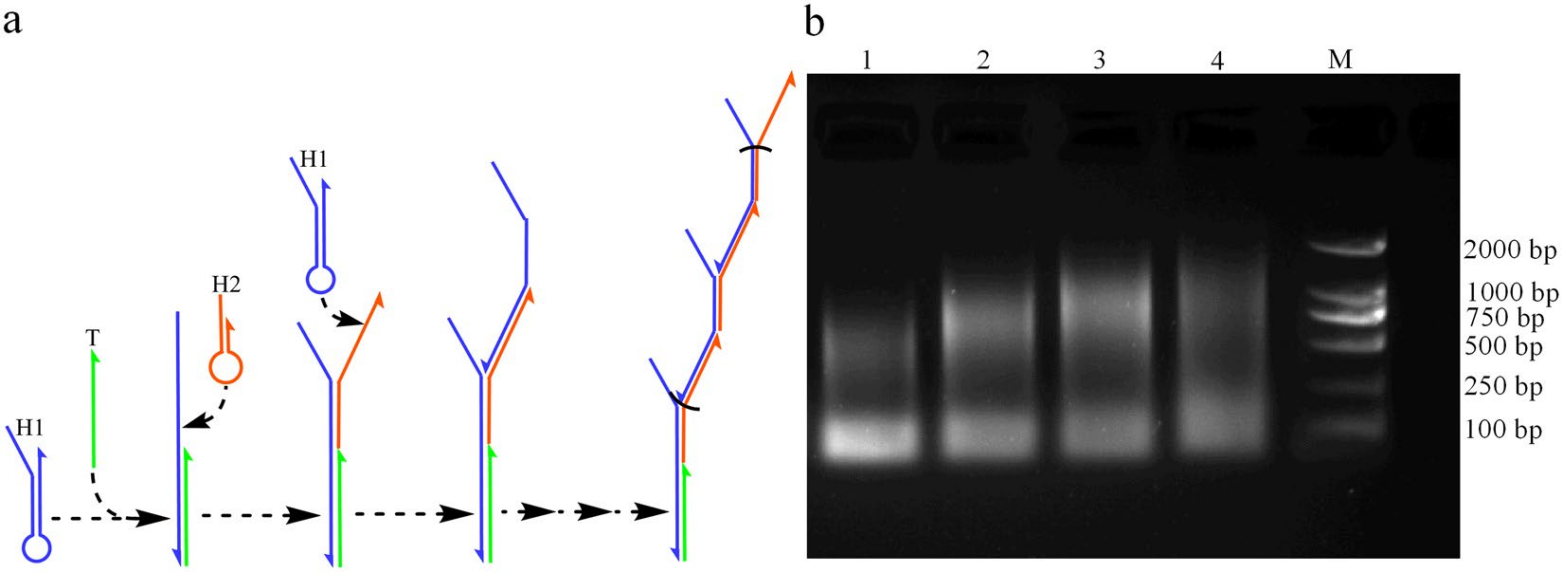
(**a**) The principle of basic HCR system. H1 and H2 were two stable species of DNA hairpins, and T was a single-stranded DNA target used as an initiator. (**b**) Agarose gel electrophoresis of HCR products. Each reaction was 10 μL (10 mM PBS, 5 mM MgCl_2_, pH 7.4) and incubated for 15 min at room temperature. Lane 1. 0.5 μM H1 + 0.5 μM H2 + 0.1 μM target DNA; Lane 2–4. 10%, 20%, 40% PEG 200 were added on the basis of Lane 1, respectively; Lane M. DL 2000 bp DNA Marker.

Firstly, we explored the effect of PEG 200 on linear DNA strand displacement reaction. HCR^12^ is a classical, enzyme-free, and linear growth hybridization chain reaction based on two stable species of DNA hairpins and DNA strand displacement reaction (Fig. 3a). In order to verify the effect of PEG 200 on HCR, the different concentrations of PEG 200 were added to HCR reaction system. HCR products were analyzed by agarose gel electrophoresis (Fig. 3b). It was seen that the yield and molecular weight of HCR products in the presence of 10% or 20% PEG 200 (Fig. 3b, Lane 2–3) were obviously superior to that of PEG-free HCR reaction (Fig. 3b, Lane 1). Thus, we could conclude that 10% and 20% PEG 200 indeed catalyzed HCR and the rate constant of HCR added 20% PEG 200 was about 100 folds compared with that of PEG 200-free HCR (Supplementary Figure S11). The positive effect of PEG 200 on HCR confirmed that the substance with increasing dissociation rate constant K2 could accelerate DNA self-assembly reaction. However, there were only a few of products when the concentration of PEG 200 was increased to 40% (Fig. 3b, Lane 4), which demonstrated HCR was inhibited in the presence of high concentration of 40% PEG 200. In addition, we also verified that betaine with decreasing association rate constant K1 indeed inhibited HCR (Supplementary Figure S12). Conversely, PEG 200 with increasing dissociation rate constant K2 improved HCR.

### The effect of PEG 200 on exponential hairpin assembly

**Figure 4:**
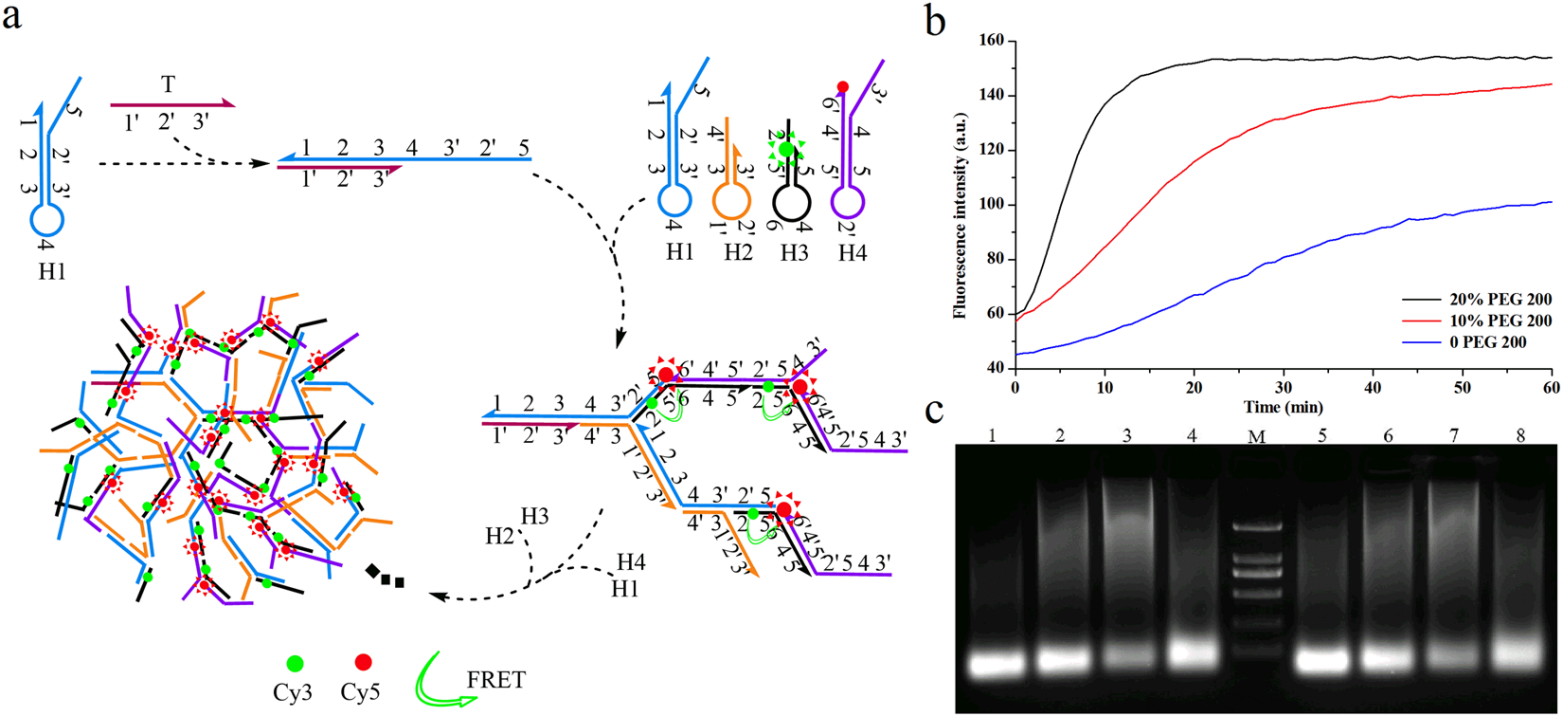
(**a**) The principle of exponential hairpin assembly (EHA). H1, H2, H3 and H4 were four stable species of DNA hairpins for EHA reaction, and T was target DNA. (**b**) The effects of different concentrations of PEG 200 on EHA reaction by real-time fluorescence. (**c**) Agarose gel electrophoresis of EHA products. Lane 1. H1 + H2 + H3 + H4 + 10 nM target DNA; Lane 2–4. 10%, 20%, 40% PEG 200 were added on the basis of Lane 1, respectively; Lane M. DL 2000 bp DNA Marker, Lane 5. H1 + H2 + H3 + H4 + 1 nM target DNA; Lane 6–8. 10%, 20%, 40% were added on the basis of Lane 5, respectively.

We next assessed the effect of PEG 200 on exponential hairpin assembly (EHA)^16, 17^ (Fig. 4a). EHA reaction employed four hairpins H1, H2, H3 and H4. The Cy3 and Cy5 were respectively conjugated to hairpins H3 and H4. The Cy3 and Cy5 were separated in the absence of target DNA. When target DNA was present, Cy3 conjugated to hairpin H3 was brought close to Cy5 on the neighboring hairpin H4, and the energy was transferred from Cy3 to Cy5. Thus, the EHA reaction could be detected by observation of the lasing emission from Cy5 at approximate 660 nm^16^.

To investigate the effect of PEG 200 on exponential DNA assembly, 10% and 20% PEG 200 were respectively added to EHA system. As shown in Fig. 4b, the fluorescence intensity was rapidly increased in a short time in the presence of PEG 200. EHA reaction rapidly reached a plateau about 20 min when 20% PEG 200 was added to EHA system. In contrast, the fluorescence intensity was still mounting for 60 min in PEG 200-free EHA reaction. The result of agarose gel electrophoresis for EHA reaction products has also proved it (Fig. 4c). We could see that the yield and molecular weight of EHA products in the presence of 10% or 20% PEG 200 (Fig. 4c, Lane 2–3 and 6–7) were larger than that of PEG-free EHA reaction (Fig. 4c, Lane1 and 5). Therefore, PEG 200 indeed accelerated EHA reaction and greatly improved its reaction efficiency. The rate constant of EHA was determined by the reaction equation as reported previously^27^. The rate constant of EHA added 20% PEG 200 was about 10 folds compared with that of PEG 200-free EHA. According to the literatures^19, 20, 23, 26^, molecular crowding and hydrophobic interactions created by PEG 200 might be important factors for DNA self-assembly.

### The effect of DMSO on HCR

It has been reported that PEG and DMSO were usually employed as enhancers in PCR to template with high GC content^21–24^. Therefore, we further verified the effect of DMSO on DNA self-assembly. The different concentrations of DMSO were added to HCR reaction system and HCR products were analyzed by agarose gel electrophoresis (Supplementary Figure S13). According to HCR products provided on agarose gel electrophoresis, although DMSO was also able to catalyze DNA self-assembly in certain concentrations, the effect of DMSO on HCR were not approaching that of PEG 200 (Fig. 3b, Lane 2–3) and this result was consistent with that of Fig. 2.

## Conclusions

DNA has indeed been employed as a versatile molecule for engineering DNA nanostructures. Currently, DNA self-assembly is usually driven by the energy of base-pair formation for hybridization-based systems or entropy-driven^11^. Thus, the enzyme-free DNA self-assembly have shown promising potential to be widely used for its simplicity, which is also one of main advantages of DNA self-assembly. Unfortunately, DNA self-assembly did not offer sufficient reaction rate and yield. How to improve the rate and yield of DNA self-assembly would be very crucial for future developments of this field. Although the engagement of enzymes in DNA self-assembly may circumvent this problem, it largely overrides the simplicity of DNA self-assembly. The vulnerability of enzymes would greatly limit the real application potential of DNA self-assembly in regulating cell functions, delivering therapeutic reagents, and so on^28, 29^.

In this work, we have found that PEG 200 with decreasing Tm of DNA and increasing dissociation rate constant K2 could accelerate the dissociation degree of dsDNA, improve the rate of DNA strand exchange, and especially catalyze DNA self-assembly to give a faster and more efficient self-assembled DNA nanostructure. The PEG-catalyzed DNA self-assembly was simpler and more robust, which was very different from the enzyme-mediated DNA self-assembly. It is envisioned that our finding will greatly spur further development in the aspects of interaction of DNA, nucleic acid amplification, and structural DNA nanotechnology.

## Electronic supplementary material

DNA Self-assembly Catalyzed by Artificial Agents
