# Supplementary material for: DNA Self-assembly Catalyzed by Artificial Agents
Source: Sci Rep. 2017 Jul 28;7:6818. doi: 10.1038/s41598-017-07210-y (PMC5533707; doi:10.1038/s41598-017-07210-y)
Supplement: Supplementary file 1 — DNA Self-assembly Catalyzed by Artificial Agents [file 41598_2017_7210_MOESM1_ESM.pdf]

Supporting Information

DNA Self-assembly Catalyzed by Artificial Agent

Chao Shi[†], Yifan Wang[‡], Menghua Zhang[‡] and Cuiping Ma[‡,*]

[†] *College of Life Sciences, Qingdao University, Qingdao, 266071, P.R. China.*

[‡] *Key Laboratory of Sensor Analysis of Tumor Marker, Ministry of Education, College of Marine Science and Biological Engineering, Qingdao University of Science and Technology, Qingdao 266042, P.R. China.*

***Corresponding author. Tel (Fax): +86-532-84022680; E-mail: mcp169@163.com**

Supplementary Figures

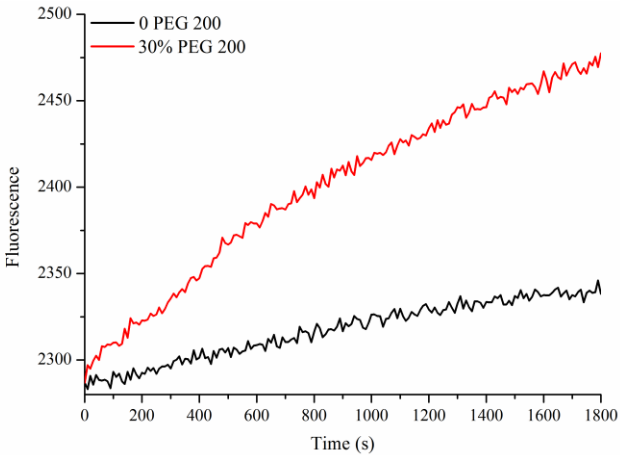

Figure S1. Real-time fluorescent unwinding of dsDNA A^FB^D with 30% PEG 200. 4.0×10^{-7} M dsDNA A^FB^D stored at -20℃ was taken out and incubated at 37℃ in 1×Thermopol Buffer (20 mM Tris-HCl，10 mM KCl，10 mM $(NH_4)_2SO_4$，2 mM $MgSO_4$ and 0.1% Triton X-100，pH 8.8) in the absence or presence of 30% PEG 200. The real-time fluorescence detection was performed using a CFX96^{TM} Real-Time PCR detection system (Bio-Rad) at 10 s intervals.

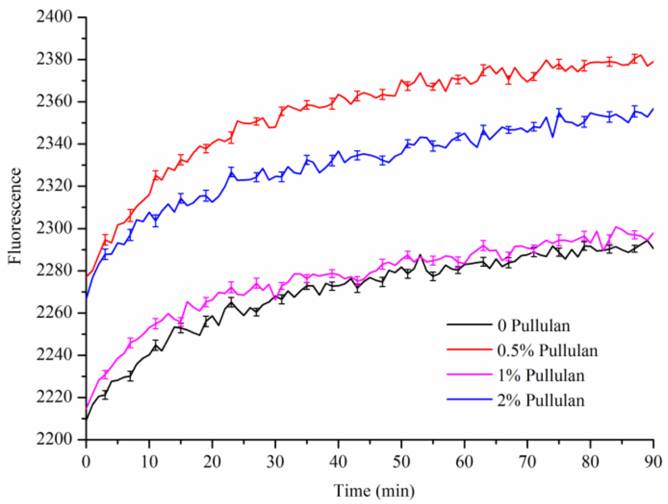

Figure S2. Real-time fluorescent DNA strand exchange with different concentrations of pullulan.

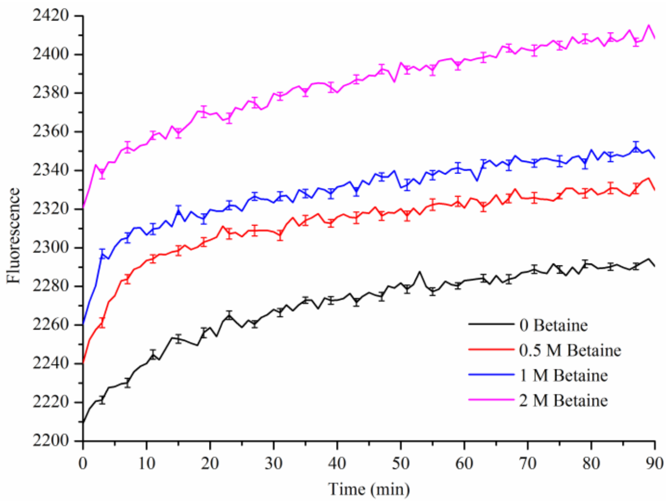

Figure S3. Real-time fluorescent DNA strand exchange with different concentrations of betaine.

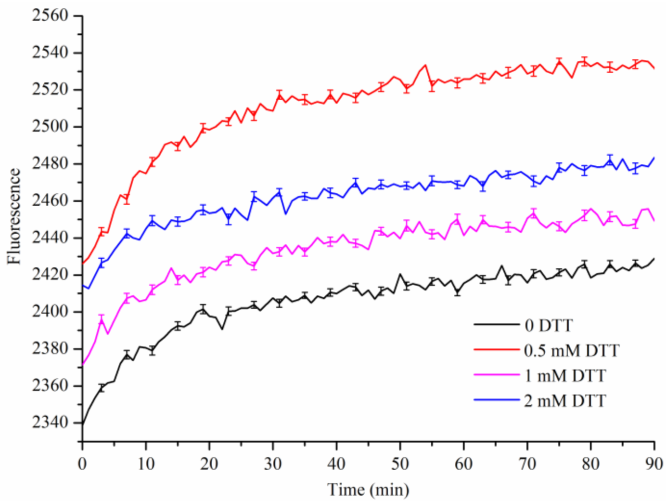

Figure S4. Real-time fluorescent DNA strand exchange with different concentrations of dithiothreitol (DTT).

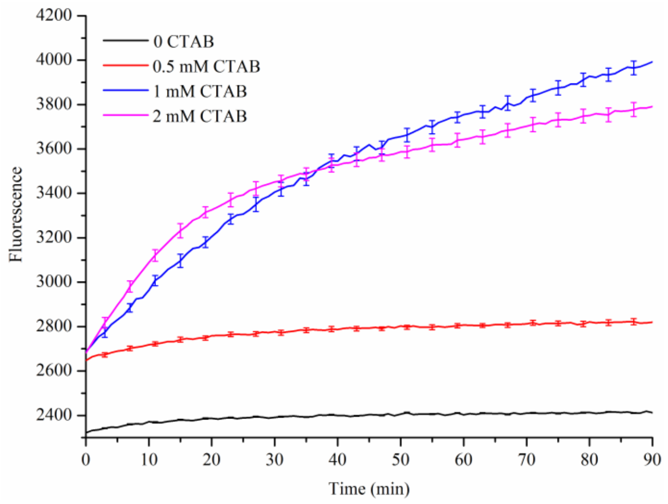

Figure S5. Real-time fluorescent DNA strand exchange with different concentrations of cetyltrimethylammonium bromide (CTAB).

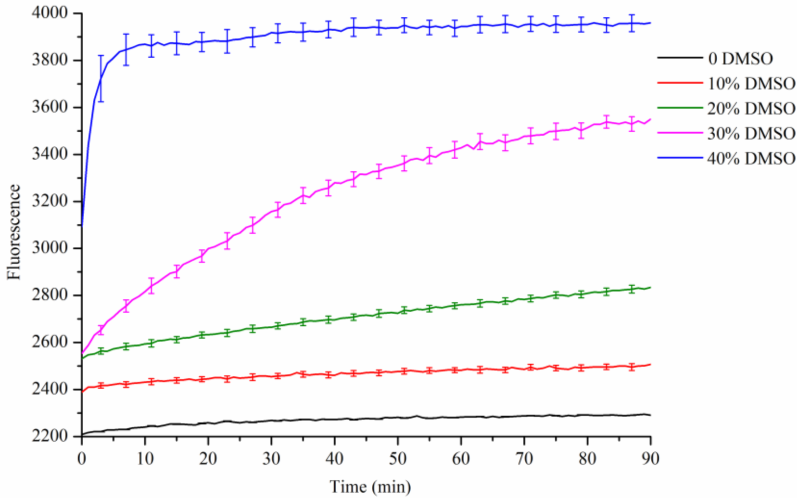

Figure S6. Real-time fluorescent DNA strand exchange with different concentrations of dimethyl sulfoxide (DMSO).

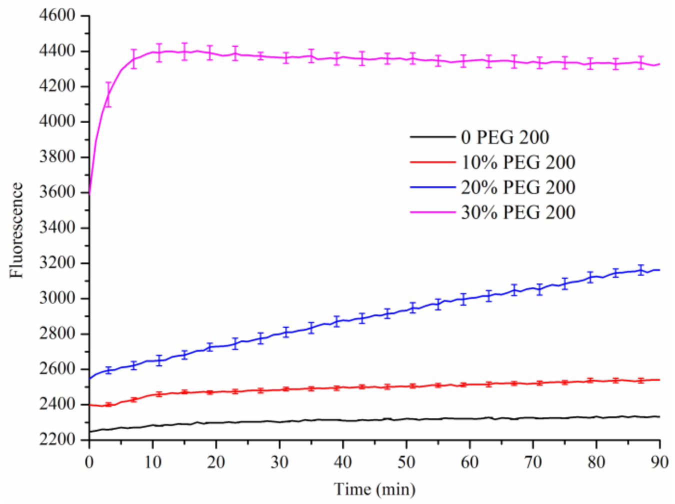

Figure S7. Real-time fluorescent DNA strand exchange with different concentrations of PEG 200.

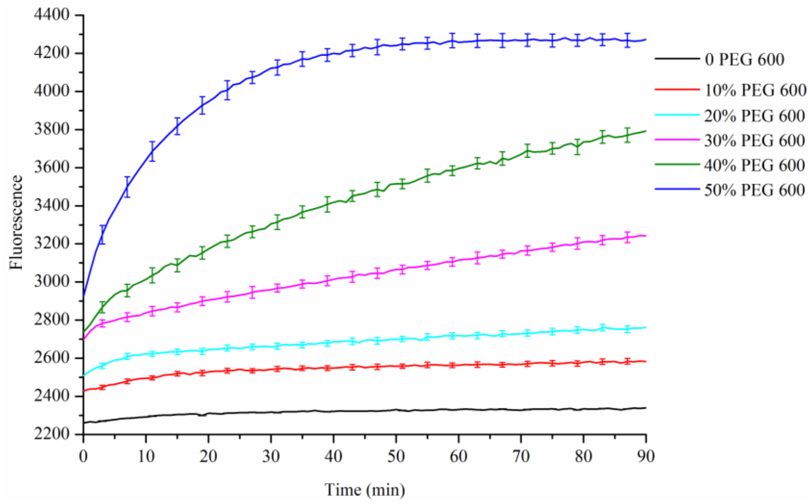

Figure S8. Real-time fluorescent DNA strand exchange with different concentrations of PEG 600.

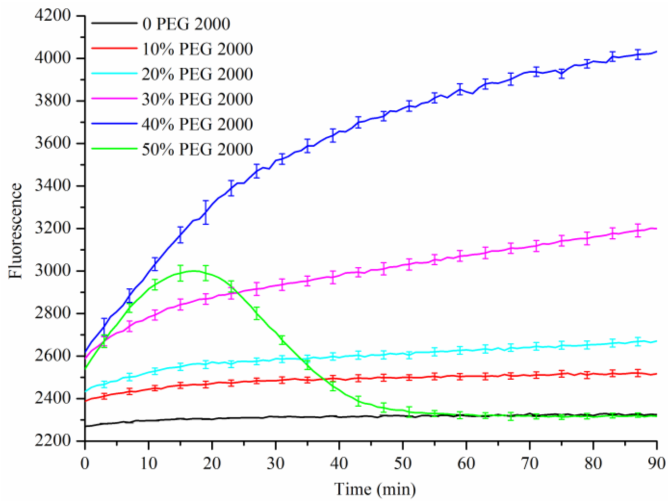

Figure S9. Real-time fluorescent DNA strand exchange with different concentrations of PEG 2000.

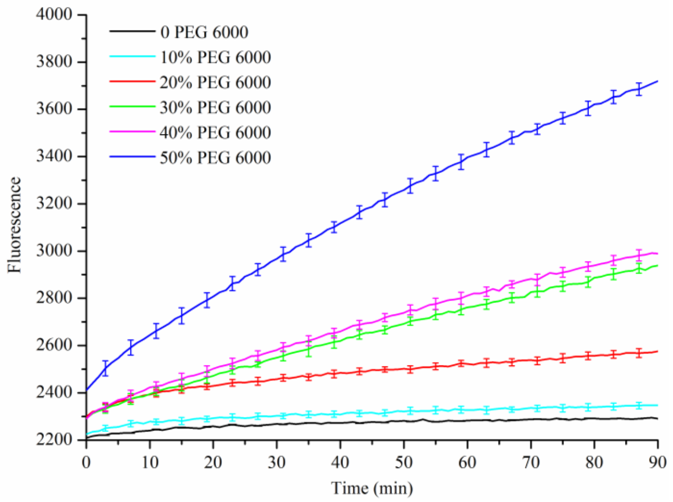

Figure S10. Real-time fluorescent DNA strand exchange with different concentrations of PEG 6000.

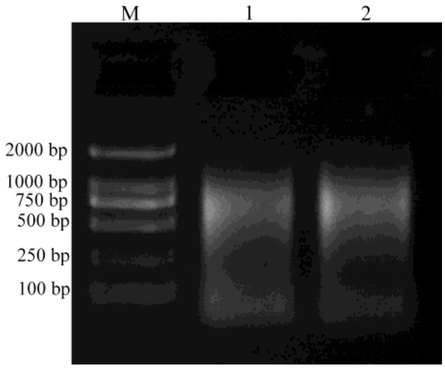

Figure S11. Agarose gel electrophoresis of HCR products with and without PEG 200. Each reaction was 10 µL and contained 0.5 µM H1 + 0.5 µM H2 + 0.1 µM target DNA. Lane 1. The reaction was incubated 15 min at room temperature with 20% PEG 200; Lane 2. The reaction was

incubated 24 h at room temperature without PEG 200; Lane M. DL 2000 bp DNA Marker.

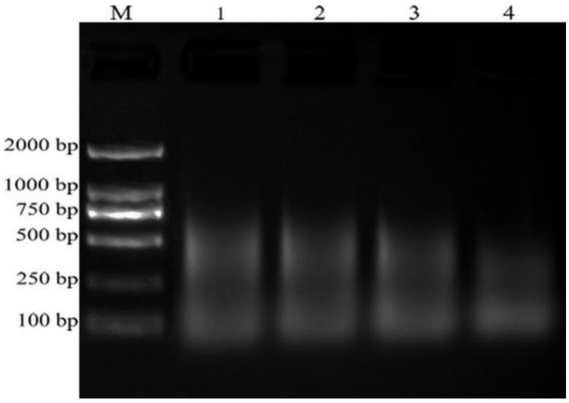

Figure S12. Agarose gel electrophoresis of HCR products with betaine. Each reaction was 10 μL and incubated 15 min at room temperature. Lane 1. 0.5 μM H1 + 0.5 μM H2 + 0.1 μM target DNA; Lane 2-4. 0.5 M, 1 M, 2 M betaine were added on the basis of Lane 1, respectively; Lane M. DL 2000 bp DNA Marker.

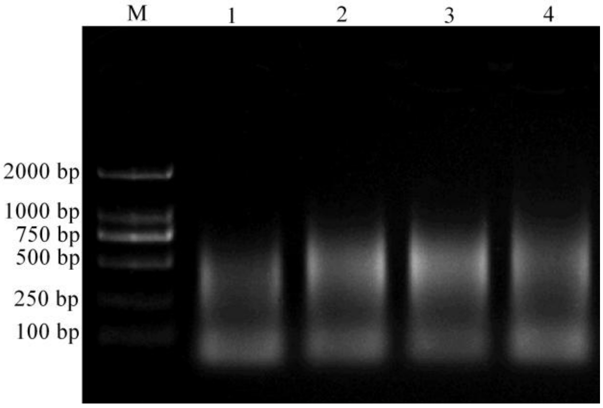

Figure S13. Agarose gel electrophoresis of HCR products with DMSO. Each reaction was 10 μL and incubated 15 min at room temperature. Lane 1. 0.5 μM H1 + 0.5 μM H2 + 0.1 μM target DNA; Lane 2-4. 10%, 20%, 40% DMSO were added on the basis of Lane 1, respectively; Lane M. DL 2000 bp DNA Marker.

Supplementary Table

Supplementary Table S1. Sequences of nucleic acids used in this work.[23-24]

Name (domain)	Sequence (from 5′ to 3′)
H1 (5-2'-3'-4-3-2-1)	CTGTGAGTGAACTGCGAG-**ACAACC-GAAACCGTTAGAGCCAAC**-*CAGAAC*-**GTTGGCTCTAACGGTTTC-GGTTGT**-GGATTG
H2 (4'-3-1'-2'-3')	GTTCTG-**GTTGGCTCTAACGGTTTC**-*CAATCC*-*ACAACC*-**GAAACCGTTAGAGCCAAC**
H3 (2-5'-6-4-5)	GGTTGT-**CTCGCAGT**(Cy3)**TCACTCACAG**-*AGGAGT*-*CAGAAC*-**CTGTGAGTGAACTGCGAG**
H4 (3'-4-5-2'-5'-4'-6')	GAAACCGTTAGAGCCAAC-**CAGAAC-CTGTGAGTGAACTGCGAG**-*ACAACC*-**CTCGCAGTTCACTCACAG-GTTCTG**-ACTCCT-Cy5
T (1'-2'-3')	CAATCC-ACAACC-GAAACCGTTAGAGCCAAC

The sequences were annotated with domain names, each of which represented a short oligonucleotide fragment. The numbered domain was complementary with the corresponding marked domain by a symbol ('). The boldface and italic portions in hairpins were stems and loops, respectively.
